# Pharmacodynamics of Memantine: An Update

**DOI:** 10.2174/157015908783769671

**Published:** 2008-03

**Authors:** G Rammes, W Danysz, C.G Parsons

**Affiliations:** 1 Clinical Neuropharmacology, Max Planck Institute of Psychiatry, 80804 Munich, Germany;; 2 Preclinical R & D, Merz Pharmaceuticals, Eckenheimer Landstrasse 100, 60318 Frankfurt am Main, Germany;; 3 Clinic rechts der Isar, Department of Anaesthesiology, Technical University, 81675 Munich, Germany

## Abstract

Memantine received marketing authorization from the European Agency for the Evaluation of Medicinal Products (EMEA) for the treatment of moderately severe to severe Alzheimer´s disease (AD) in Europe on 17^th^ May 2002 and shortly thereafter was also approved by the FDA for use in the same indication in the USA. Memantine is a moderate affinity, uncompetitive N-methyl-D-aspartate (NMDA) receptor antagonist with strong voltage-dependency and fast kinetics. Due to this mechanism of action (MOA), there is a wealth of other possible therapeutic indications for memantine and numerous preclinical data in animal models support this assumption. This review is intended to provide an update on preclinical studies on the pharmacodynamics of memantine, with an additional focus on animal models of diseases aside from the approved indication. For most studies prior to 1999, the reader is referred to a previous review [196].

In general, since 1999, considerable additional preclinical evidence has accumulated supporting the use of memantine in AD (both symptomatic and neuroprotective). In addition, there has been further confirmation of the MOA of memantine as an uncompetitive NMDA receptor antagonist and essentially no data contradicting our understanding of the benign side effect profile of memantine.

## THERAPEUTIC TARGET

The maximal therapeutically-relevant plasma concentration of memantine is around 1 µM (see [55,203]). Brain extracellular fluid (ECF) concentration of around 0.8 µM can be anticipated [102] and any receptor that expresses an affinity at, or below, the very low µM range should be considered as a potential therapeutic target. Given this assumption, there are only four plausible known target types to date: the most likely is the NMDA receptor channel, but 5-hydroxy-trypta-mine (5-HT_3_) receptors [220] and α7 and/or α4β2 nicotinic receptors [11] should also be taken into consideration [11, 37, 165].

The α7 and α4β2 nicotinic acetylcholine receptor and the 5-HT_3_ receptor systems have been suggested to play a role in modulating CNS functions, including learning and memory. These receptors are structurally related, containing large extracellular ligand-binding domains and four transmembrane domains that are largely conserved. They exhibit considerable cross-pharmacology, e.g. high concentrations of the α7 nAChR agonist nicotine inhibit 5-HT_3_ receptor-mediated responses, and high concentrations of the 5-HT_3_ receptor agonist serotonin inhibit α7 nAChR-mediated responses. Based on this knowledge, it seems reasonable to investigate whether ligands specific for either receptor, might have affinity for both. 

## NMDA RECEPTORS

### Fast Kinetics and Strong Voltage-Dependency

Memantine blocks the NMDA receptor channel in an use-dependent manner, meaning that it can only gain access to the channel in the presence of agonist and remains largely trapped in the channel following removal of agonist [113, 196, 198]. Both the clinical tolerability and symptomatic effects of memantine have been attributed to its moderate affinity (IC_50_ around 1µM at –70 mV) and associated fast blocking / unblocking kinetics and strong voltage-depen-dency [113, 196, 198, 229]. These properties have been characterized and confirmed by numerous groups using whole cell patch clamp recordings from primary cultures of hippocampal and cortical neurons as well as for NMDA receptors expressed heterologously in HEK-293 cells [29, 34, 43, 154, 198-201, 259, 260]. How these biophysical properties account for the better therapeutic safety of memantine compared to other channel blockers such as (+)MK-801 and phencyclidine has been a matter of considerable debate and there are several theories.

Memantine and other well tolerated open channel blockers show much faster open channel blocking / unblocking kinetics than compounds burdened with negative psychotropic effects such as (+)MK-801 or phencyclidine [28, 28, 44, 196, 198, 229, 231]. The kinetics of (+)MK-801 and phencyclidine are too slow to allow them to leave the channel upon depolarization, which is often reflected in apparently weaker functional voltage-dependency. These two parameters are directly related to affinity, with lower affinity compounds such as memantine showing faster kinetics and apparently stronger voltage-dependency, as reflected in an estimated δ value of around 0.8 [201]. The δ value describes the percentage of the trans-membrane field the drug experiences when blocking the NMDA receptor channel [196]. The unblocking rate of memantine in the continuous presence of this antagonist following depolarizing voltage-steps is very rapid and well within the time course of NMDA receptor-mediated EPSP. 

Memantine blocks and unblocks open NMDA receptor channels with double exponential kinetics. The amplitude and speed of the fast component of block increases with memantine concentration. In contrast, the speed of fast unblock remains constant but its weight (relative to the slow component) decreases with memantine concentration [29, 34, 77, 259, 260]. Moreover, the predominant effect of depolarization is to increase dramatically the weight of the faster recovery time-constant [34, 77, 199]. These data indicate that memantine binds to at least two sites within the channel [259, 260].

### Fast Agonist Concentration-Dependent Access to the Open Channel During Pathological Activation

It has been proposed that the ability of low affinity open channel blockers to gain rapid access to the NMDA receptor channel is important in determining their therapeutic safety in ischemia and epilepsy [44, 229, 231]. However, this hypothesis alone cannot explain the better therapeutic profile of memantine in AD as, even if receptors were only blocked following pathological activation, most would then remain blocked in the continuous presence of memantine and therefore be unavailable for subsequent physiological activation - in the therapy of AD, memantine is continuously present at very stable steady-state concentrations due to its very long half life in humans.

It has further been suggested that memantine blocks NMDA receptor channels in an agonist concentration-depen-dent manner i.e. the more agonist, the more equilibrium blockade [152]. This assumption is supported neither by the blocking model proposed by [29] nor by our own recent unpublished data which highlight, for us, the technical problems in performing such studies [86]. At very low agonist concentrations, the open channel probability is very much reduced and open channel blockers take much longer to access and to leave the open channel. As such, it seems most likely that memantine blockade only appeared to be less efficacious at lower agonist concentrations in previous publications because it had not really reached equilibrium blockade.

### Fast, Voltage-Dependent Unblock from the Open Channel During Synaptic Activation–Basis for the Signal-to-Noise Hypothesis

Physiologically, NMDA receptors are transiently activated by mM concentrations of glutamate [48] following strong depolarization of the postsynaptic membrane, which rapidly relieves their voltage-dependent blockade by Mg^2+^ [189] whereas, during pathological activation, NMDA receptors are activated by lower concentrations of glutamate but for a much longer time [9, 19, 36, 87, 88, 177]. The voltage-dependency of the divalent cation Mg^2+^ is so pronounced that it also leaves the NMDA channel upon moderate depolarization under pathological conditions, i.e. in the presence of tonically mildly elevated glutamate levels and moderate membrane depolarization due to energy deficits. Although uncompetitive antagonists also block the NMDA receptor channel, high affinity compounds such as (+)MK-801 have much slower unblocking kinetics than Mg^2+^ and less pronounced functional voltage-dependency and are therefore unable to leave the channel within the time course of a normal NMDA receptor-mediated excitatory post-synaptic potential. As a result, (+)MK-801 blocks both the pathological and physiological activation of NMDA receptors [196].

We were the first to suggest that the combination of fast offset kinetics and strong voltage-dependency allows memantine to rapidly leave the NMDA channel upon transient physiological activation by mM concentrations of synaptic glutamate, but block the sustained activation by µM concentrations of glutamate under moderate pathological conditions [198, 200, 201]. This hypothesis is further supported by the fact that, although the slower component of offset kinetic at near resting membrane potentials and room temperature is still too slow to allow synaptic activation - i.e., around 5 seconds, the relief of blockade in the continuous presence of memantine upon depolarization is much faster due to an increase in the weight of the faster recovery time-constant [34, 77, 259, 260]. These kinetics are almost certainly even faster *in vivo* due to higher temperatures [57]. Furthermore, the rate of recovery from memantine blockade is dependent on the open probability of NMDA channels [43, 197] and therefore would be faster in the presence of higher, synaptic concentrations of glutamate [48].

Given the crucial role of NMDA receptor synaptic activation in neuronal plasticity, simplistic interpretation of the observation that a NMDA receptor antagonist such as memantine can improve cognition and neuronal plasticity under pathological conditions might seem to be paradoxical. It should, however, be stressed that divalent cation Mg^2+^ is an endogenous NMDA receptor channel blocker and its absence leads both to an impairment in neuronal plasticity [49, 74] and neuronal death [81]. Any dysfunction of postsynaptic neurons leading to weakened blockade by Mg^2+^, e.g. due to partial depolarization as a consequence of an energy deficit, may also trigger prolonged Ca^2+^ influx and structural (neuronal loss) deficits [54, 230]. Because memantine is more potent and slightly less voltage-dependent than Mg^2+^ due to its simple monovalent charge, it may thus serve as a more effective surrogate for Mg^2+^ [198]. As a result of its somewhat less pronounced functional voltage-dependency, memantine is more effective than Mg^2+^ in blocking tonic pathological activation of NMDA receptors at moderately depolarized membrane potentials. However, following strong synaptic activation, memantine, like Mg^2+^, can leave the NMDA receptor channel with voltage-dependent, fast unblocking kinetics. In turn, memantine not only suppresses tonic pathological synaptic “noise” but also allows the relevant physiological synaptic signal to be detected. This provides both neuroprotection and symptomatic restoration of synaptic plasticity by one and the same mechanism [54, 196]. Antagonists that have “too high” affinity for the channel or “too little” functional voltage-dependence, such as dizocilpine ((+)MK-801), thus produce numerous side effects, since they essentially act as an irreversible plug of the NMDA receptor channel and block both pathological and physiological function. 

### Partial Untrapping

Another theory to explain the better therapeutic tolerability of memantine was proposed in a seminal paper by [29] and supported by data from [169, 259] (see [113] for review). Their data indicate that memantine and amantadine appear to have a lesser tendency to be trapped in NMDA receptor channels than do phencyclidine or (+)MK-801. This difference was attributed to the ability of channel blockers to increase the affinity of NMDA receptors for agonist and the faster kinetics of the amino-adamantanes. Receptors blocked by memantine retain agonist and thereby open and release memantine following removal of both agonist and memantine from the extracellular solution - see also [43]. This partial untrapping was reported to be less pronounced for higher affinity compounds as their slower unblocking kinetics do not allow them to leave the channel quickly enough following agonist removal.

Recently, Dr. Johnson’s group provided more data characterizing the mechanism underlying partial trapping of memantine but not ketamine in the NMDA receptor channel [113, 129]. Their most recent data are also consistent with a model in which there are two binding sites for memantine, a deep site inside of the external gate that allows trapping and a shallow site outside the gate that does not (see also [32]. Ketamine only binds to the deep site [113, 129]).

This same group [29] proposed that partial trapping may underlie the better therapeutic profile of memantine, as a proportion of channels - around 15 to 20% - would always unblock in the absence of agonist and thereby be available for subsequent physiological activation. In other words, the antagonism by memantine is like that of a low intrinsic activity partial agonist in that it does not cause 100% blockade of NMDA receptors. However, although this theory can be used to explain the therapeutic tolerability of memantine, it provides no MOA for the symptomatic effects on cognition observed in AD patients.

### Channel Blocking Site

Several studies indicate that memantine and Mg^2+^ block at the same or similar channel site as they are mutually exclusive - as evidenced by the kinetics of unblock in the presence of both [44, 259]. Moreover, the potency of memantine was observed to be reduced 20 fold by mutations at the N-site (N598Q) of the M2 trans-membrane segment in NR1 subunits and 30- to 100- fold by double mutations (W593L/ N598Q) within the channel-forming domain. Double mutations at the equivalent L- (L577W) and Q/R-sites (Q582T) in GluR1 receptors permitted open channel blockade of AMPA receptors by memantine (IC_50_ at -70 mV = 1.3 µM, δ=0.75) [71]. In this study on human NR1/NR2A receptors expressed in *Xenopus* oocytes, memantine blocked normal receptors in a strongly voltage-dependent manner (IC_50 _at -80 mV= 0.3 µM, δ = 0.77; [71]). 

In a more recent study in *Xenopus *oocytes, memantine blocked rat NR1a/2B receptors with an IC_50_ of 0.46 µM at –70 mV. The effects of memantine were strongly voltage-dependent with a δ value of 0.89 and a Kd(0) value of 7.1 µM. Mutations at the critical asparagine residues in the M2 loop of NR1 and NR2B and at a tryptophan residue in M2 of NR2B reduced blocking by MK-801, memantine and TB-3-4. In contrast, many mutations at residues in the pre-M1, M1, M3, post-M3 and post-M4 regions reduced blocking by MK-801 and TB-3-4, but had no effect on the block by memantine [116].

We attempted to verify the common channel blocking locus proposed for memantine and Mg^2+^ using channel mutations at the N-sites of NR1 and NR2 receptor subunits [202]. N598G mutations at the N-site of the M2 trans-membrane segment in NR1a subunits and N595G or N596G mutations in the NR2A subunit had little or no effect on the potency of memantine. These same mutations had pronounced effects on the potency of Mg^2+ ^and double mutations may have even apparently permitted permeation of this divalent cation[117, 299, 300]. This contrasts with the N598Q mutation data [71] which decreased the potency of memantine but had little or no effect on Mg^2+^ voltage-dependent block [237, 299]. These differences gave important insights into differences in the mechanism of channel blockade by memantine and Mg^2+^.

All mutations in our own internal study replaced asparagine residues with short “naked” residues. These sites are known to act as channel binding sites for Mg^2+^ and these mutations, as expected, reduced the potency of Mg^2+^ [299]. In contrast, they did not have any effect on the potency of memantine, indicating that memantine can still plug the widened pore (from 0.55 to 0.7 nm). The N598Q mutation used by [71] replaces an asparagine with a longer glutamine residue. This could reduce the pore diameter and thereby prevent memantine from penetrating so deeply whilst having little effect on the Mg^2+^ binding site. This difference indicates that, in contrast to Mg^2+^, the channel block by memantine is determined largely by the size of the channel pore. 

In contrast to glycine site NMDA receptor antagonists, memantine (12 µM) did not inhibit NR1/NR3 “excitatory glycine” receptors expressed in *Xenopus* oocytes [42].

### *In Vivo* NMDA Receptor Antagonism

Memantine selectively reduced responses of single spinal neurones to micro-iontophoretic application of NMDA with an ID_50_ of around 2 mg/kg i.v. in anaesthetised rats [184] and inhibited NMDA-induced convulsions in mice with an ID_50_ of 4.6 mg/kg i.p. [27, 201]. In a more recent study, memantine protected against NMDA-induced convulsions (ED_50_ 2.9 mg/kg) at doses that were 7-, 8- to 18-fold lower than those producing effects on MK-801 discrimination, ataxia or locomotion respectively [85]. In rats, convulsions produced by i.c.v. injection of NMDA were inhibited by memantine with an IC_50_ of 9.7 mg/kg [27]. Memantine was also potent with an ID_50_ of 2.7 mg/kg against NMDA-induced damage of cholinergic neurons in the Nucleus Basalis of Meynert (NBM) [288].

Initial data indicated that much higher doses of memantine were required to block responses of spinal neurones to micro-iontopho-retic NMDA (ID_50_ of 26 mg/kg i.v.) [100]. However, more recent data from the same group [114, 167], showed memantine to be more potent when the control NMDA responses were of low intensity. Memantine (10 mg/kg), ketamine (2 mg/kg) and (+)MK-801 (0.1 mg/kg i.v.) reduced responses to similar levels (15-25% of control). Memantine was less effective against stronger intensity responses of the same neurons and this difference was reflected in a strong correlation between ID_50_ and control firing rate. (+)MK-801 was equieffective and ketamine was somewhat less effective against stronger responses. This difference likely reflected the stronger voltage-dependency of memantine and probably underlies the differences observed in the *in vivo* potencies of memantine in different laboratories (see [100, 184]).

### 5-HT_3 _Receptors

5-HT_3_ receptors are also ligand-gated ionotropic receptors permeable for monovalent cations. 5-HT_3_ receptors not only have a high density in the area postrema but also in the hippocampal and amygdala regions of the limbic system. The immediate consequence of neuronal depolarization resulting from 5-HT_3_ receptor activation is neurotransmitter release, particularly dopamine in mesolimbic pathways, which suggests a potentially important role for this receptor system in neuronal circuitry involved in drug abuse. As such, 5-HT_3 _receptor antagonists may, in addition to their well characterized efficacy in emesis and irritable bowel syndrome, also be useful in the treatment of drug abuse, anxiety, cognitive deficits and depression [51, 90, 93, 128, 155, 155, 166, 210, 210].

We investigated the effects of memantine, amantadine and neramexane on human 5-HT_3_ receptors stably expressed in HEK-293 cells and on native murine 5-HT_3 _receptors in the N1E-115 cell line [220]. Our results clearly showed that memantine, neramexane and other amino-alkyl-cyclohexanes are, in addition to their well characterized action as uncompetitive NMDA receptor antagonists, also antagonists of 5-HT_3_ receptors. These effects were seen at concentrations similar to those required for uncompetitive antagonistic effects at NMDA receptors. These uncompetitive NMDA receptor antagonists had similar effects on 5-HT_3_ receptors to those previously reported for different classes of anti-depres-sants [69] and antipsychotics [218] i.e., they probably antagonized responses by inducing desensitization. The effects were not voltage- or use-dependent and there was no evidence for open channel blockade. Importantly, similar effects were not seen with ketamine.

Moreover, it seemed possible that combined antagonistic effects at NMDA and 5-HT_3_ receptors by e.g. memantine would lead to positive synergistic effects, which could contribute to the therapeutic safety and efficacy in AD by increasing desired effects - cognitive enhancement [294] and antidepressive activity [69, 70, 293] whilst further reducing possible negative effects of NMDA receptor antagonism e.g. by reducing mesolimbic dopamine hyperactivity [93].

However, antagonism of the 5-HT_3_ receptor does not seem to play a major role for the good tolerability of memantine versus e.g. ketamine *in vivo* in rats. Thus, recent data indicate that 5-HT_3_ receptor blockade with MDL 72222 (0.3-3 mg/kg) does not ameliorate the behavioural deficits (ataxia, stereotypes, deficits in prepulse inhibition and learning) of ketamine [127] - an NMDA receptor antagonist that does not itself block 5-HT_3_ receptors [220]. However, these authors proposed that 5-HT_3_ antagonism might still contribute to certain effects, such as the antidepressant-like actions [127]. Memantine (5 mg/kg) had no anti-emetic effect in cisplatin-treated ferrets, in contrast to treatment with odansetron or granisetron [146], indicating a lack of 5-HT_3_ receptor blockade after a behaviorally active dose. One caveat here, however, is that we have little information on the pharmacokinetics of memantine in this species.

### Neuronal Nicotinic Receptors

Changes in neuronal nicotinic receptors have been implicated in a number of diseases [163, 208, 238, 244, 287]. These include AD, Parkinson's disease, Tourette´s syndrome, schizophrenia, drug abuse and pain. Based on the observation that the nicotinic agonist nicotine itself seems to have beneficial effects, most drug development effort so far has aimed at the discovery of selective nicotinic agonists, especially for α4β2 receptors. On the other hand, it is unclear whether the effects of nicotinic agonists in e.g. Tourette´s syndrome and schizophrenia are due to the activation or inactivation / desensitization of neuronal nicotinic receptors - e.g. meca-mylamine is also active [239].

The effects of agonists on neuronal nicotinic receptors are strongly dependent on the exposure period. Rapid reversible desensitization occurs in milliseconds, rundown occurs in seconds, irreversible inactivation of α4ß2 and α7 containing receptors occurs in hours and their upregulation occurs within days. In other words, the effects of nicotinic "agonists" may in fact be due to partial agonism, inactivation and/or desensitization and adaptive changes in the expression of neuronal nicotinic receptors. In turn, moderate concentrations of neuronal nicotinic receptor channel blockers could produce the same effects as reported for nicotinic agonists in the above mentioned indications. This is especially likely for α7 receptors, which show complete desensitization.

As an example, there is considerable evidence that nicotinic antagonists might produce similar effects to agonist in animal models of learning. Thus, under certain conditions, both nicotine and the nicotinic antagonist mecamylamine improve learning [148]. Although nicotine improves memory, β2-deficient mice actually show improved learning and show no additional beneficial effects of nicotine [209]. *In vitro* experiments also indicate that both the activation of non-α7 nAChRs and inactivation of α7 nAChRs contribute to LTP induction [80]. Moreover, chronic nicotine-mediated facilitation of LTP induction was probably due to chronic nicotine-induced desensitization of α7 nAChRs [80]. Similarly, chronic treatment with both nicotine and mecamylamine increased nicotinic receptor number [205, 206] and both nicotine and mecamylamine enhanced haloperidol-induced catalepsy [149].

Memantine has been found to inhibit α4β2 responses with an IC_50_=6.6 or 11.3 µM [37, 63], however, this level is probably too high to be of real therapeutic significance. In line with this, *in vivo* memantine at 10 mg/kg had only mild effect on nicotine discrimination [30, 305] mediated by α4β2 receptors [162] at doses that also affected saline responding indicating lack of selectivity. The reported potency of memantine at α7 receptors varies considerably, from 0.33-1.68 µM seen in rat receptors [11, 63], to 5µM observed in human receptors [165], the latter finding making it seem less likely to be of therapeutic relevance for AD patients. Moreover, even in rats, recent studies (Nagel, personal communication) indicate that at 5-10 mg/kg, memantine does not produce changes in dopamine levels in the striatum that would be consistent with the blockade of α7 receptors [222] – in fact, memantine caused an increased release of dopamine, not a decrease as would be expected for an α7 antagonist. These results were confirmed by a micro-dialysis study showing that memantine (20mg/kg) resulted in significant increases in extracellular dopamine, norepinephrine and their metabolites in cortical regions [251].

Although memantine blocks α9/α10 nicotinic receptors with similar affinity to NMDA [191], these receptors show a very discrete distribution e.g. in cochlear hair cells and this action is therefore highly unlikely to be responsible for therapeutic effects of memantine on symptoms in AD [65, 119]. In agreement with published data [191], memantine blocked α9/α10 receptors expressed in *Xenopus* oocytes at –70 mV with an IC_50_ of 1.20 µM and in a weakly voltage-dependent manner [64].

Interestingly, the effect of memantine on all tested “neuronal” nicotinic receptors was only weakly voltage-dependent, as is the case for 5-HT_3_ receptors (see above), which often share a common pharmacology with neuronal nicotinic receptors and implies a site of action, which is not deep inside the channel.

### Summary

Taken together with data reviewed previously [55, 124], it seems clear that NMDA receptor antagonism is the primary mechanism of action of therapeutic relevance for memantine although additional effects at 5-HT_3_ receptors and neuronal nicotinic receptors may be supportive for therapeutic tolerability. The effects on NMDA are far more consistent across species i.e., human and rat, whereas effects on neuronal nicotinic receptors seem to be species-dependent and findings specific for neuronal nicotinic receptors from rodent studies may bear little or no relevance for the human therapeutic situation. Additional support for NMDA receptor antagonism *in vivo* at therapeutically-relevant doses is provided by its strong neuroprotective activity in animal models discussed later.

## PRIMARY PHARMACODYNAMICS

AD is the most common form of dementia, the other two major forms being vascular dementia (VD) and mixed Alzheimer's / vascular dementia (AD/VD). Acetylcholine esterase inhibitors (AChEi) aim to improve cognitive and symptomatic behavioral symptoms and thereby, the quality of life for the patients / caregiver. NMDA-receptor-mediated glutamate excitotoxicity is hypothesized to play a major role in both the symptoms and pathomechanism of this disease. Memantine is a clinically proven effective therapy for symptomatic treatment of Alzheimer's disease [224, 271, 297]. The preclinical data discussed below provide reason to believe that memantine, in addition to symptomatic treatment for AD, could also be an effective disease-modifying agent in this devastating disorder.

### Alzheimer’s Disease

#### Symptomatic Effects

Removal or reduction of magnesium in hippocampal slices causes deficits in LTP [49] and this could be reversed by memantine with a bell-shaped concentration-response curve with near complete reversal of this deficit by therapeutically-relevant concentrations [74]. Similarly, in hippocampal slices, low concentrations of NMDA also caused a reduction of LTP induction / expression in the CA1 region [109, 304]. This deficit was also reversed by memantine at a concentration of 1 μM [304]. In support of these effects of memantine, in knockout mice lacking the glutamate transporter GLT-1, LTP was also impaired in the hippocampal CA1 region *in vitro*. When tetanic stimulation was applied in the presence of a very low concentration of the competitive NMDA receptor antagonist AP5 (0.5 µM), the impairment was reversed [118].

Memantine (20 mg/kg/day, 7–13 days after lesioning and 4 days before training) caused a symptomatic improvement of spatial learning of rats in the radial maze following lesions of the entorhinal cortex with quinolinic acid. These memory improving effects of memantine were not related to changes in brain NGF content or choline acetyltransferase (ChAT) activity [143].

Memantine was confirmed to improve learning in a radial water maze task where rats were given 4 trials and then tested 1 h later and 1 day later. Only one arm was reinforced (safe platform) and the start on each trial was from a different position. Memantine (at 5 and 7.5 but not 2.5 mg/kg), administered 30 min before day 1 training, did not affect performance on day 1 but enhanced learning (decreased the number of errors) 24 h later. This result was explained by tempering over-activation of NMDA receptors resulting from massive glutamate release in stressful conditions such as in the water maze [310]. Withdrawal from chronic (6 month) alcohol ingestion produced robust learning deficits in rats in the Morris water maze. Treatment with memantine starting at the beginning of the withdrawal phase (20 mg/kg bolus followed by 1 mg/kg every 12 h for 4 weeks) resulted in a complete reversal of these behavioral impairments [158].

Memantine 10 mg/kg re-instated experience dependent place field neuronal firing field expansion in aged rats. This effect was claimed to be due to a dampening of Ca^2+^ overload by memantine, which fits perfectly to the signal-to-noise hypothesis [39].

Even in much lower species, memantine has been claimed to improve learning by reversing noise at glutamatergic synapses. The glutamate transporter inhibitor (L-trans-2,4-PDC)-induced olfactory amnesia in honeybees (*Apis mellifera*) was 'rescued' by memantine (1-10mM, 1µl per bee) injected either before training, or before testing, suggesting that memantine restores memory recall rather than memory formation or storage. When injected alone, memantine also had a mild facilitating effect on memory. In contrast, MK-801 also impaired memory in all tests [254].

Memantine (30 mg/kg/day; p.o.) for 2-3 weeks significantly improved the acquisition of the water maze task in APP/PS1 mice without affecting swimming speed. Memantine affected neither locomotor activity nor aggressive behavior in transgenic or wild type mice. These data indicate that memantine improves hippocampus-based spatial learning in a transgenic murine model of AD without producing non-specific effects on locomotion/exploratory activity [174].

Low doses of memantine (2 mg/kg i.p. daily for 1 week) had mild symptomatic effects on cognition in the Morris water maze in APP23 mice, especially during the probe trial. Higher doses of 10 mg/kg were ineffective. This study compared memantine to AChE inhibitors, where a consistent biphasic dose-response curve was also observed – low doses were shown to be effective, but higher doses were not. However, the difference in doses of the AChE inhibitors was two-fold, but for memantine it was 5-fold. Biphasic effects on cognition / synaptic plasticity are well known for memantine and it seems likely that better effects would have been seen had 5 mg/kg also been tested [277].

Zhao *et al*. discussed *in vitro *data to support the beneficial actions of galanthamine and memantine combinations [309]. Memantine was reported to block extrasynaptic NMDA receptors (IC_50_ = 22 nM) 100 times more potently than the synaptic NMDA receptors at negative membrane potentials (IC_50_ = 2.5 µM) and the block of both types of NMDA receptor was strongly voltage-dependent. Galanthamine (1µM) potentiated NMDA receptors in a non voltage-dependent manner. Co-application of memantine with galanthamine prevented the galanthamine potentiation and the activation of extrasynaptic NMDA receptors, but membrane depolarization revealed the galanthamine potentiation of proposed synaptic receptors. The authors therefore hypothesized that, in the presence of both drugs, cell death is expected to be prevented by memantine near the resting potential, while NMDA-mediated synaptic transmission would be further potentiated by galanthamine. These results were claimed to provide an *in vitro *basis for the beneficial actions of galanthamine and memantine combinations [309].

It has been shown *in vitro* that memantine does not attenuate the effects of AChE inhibitors used clinically in AD such as donepezil, tacrine or galanthamine, in contrast to clear attenuation of irreversible inhibition by DFP [291]. In an *in vivo* study, memantine (10mg/kg) did not inhibit AChE in any brain areas and did not interact with donepezil and rivastigmine [92]. This lack of negative interaction is also supported by recent studies investigating AChE activity in man and in clinical studies focusing on symptomatic effects [59a,^ 67^,^ 207^].

#### Neuroprotection

##### Introduction

Wenk *et al*. have published many studies indicating that memantine is neuroprotective against various insults to the cholinergic NBM. These effects were expressed both in histological and functional terms [290]. Inflammatory changes are closely related to the cognitive and neuropathological manifestations of AD [3, 4]. Infusion with lipopolysaccharide (LPS, a component of Gram-negative bacteria cell walls) led to a massive inflammatory reaction followed by cell loss in the NBM. Memantine, (20 mg/kg/day) infused s.c. in parallel to LPS, provided significant protection of NBM neurons [290, 292, 296]. 

In conformation of these positive findings, following infusion of ibotenic acid into the NBM of rats, acute administration of memantine (10 mg/kg, i.p.) blocked the increase of astrocytes and microglia activation in the cortex [2]. In a recent study from Wenk´s group, memantine did not prevent the neurotoxic effects of TNF-alpha on cholinergic neurons [289]. This, taken together with the previous studies described above, indicates that NMDA receptors are involved in neurodegeneration resulting from inflammation produced by LPS, but not TNF-alpha infusion. In this study, TNF-alpha was infused for a prolonged time, which contrasts to the short lasting increase of this cytokine following treatment with LPS as in the previous work. 

Systemic oral administration of memantine 2 mg/kg daily for 13 days, to rats with chronic partial AF64A-induced deprivation of cholinergic functions, improved their learning in a Morris water maze [14, 147]. This effect of memantine had a longer duration of action than the acetylcholine esterase (AChE) inhibitor donepezil [147]. Importantly, memantine shows no negative interaction *in vitro* and *in vivo* with AChE inhibitors used in the treatment of AD [67, 207, 291].

Memantine (20 mg/kg/day) was able to prevent and reverse the occurrence of learning and spatial memory deficits after cortical devascularization injury (inducing cortical cholinergic terminal loss and retrograde degeneration of the cholinergic projections from NBM) in rats [233].

In rats chronically infused into the 4th ventricle for 28 days with LPS (*via* osmotic minipump), there was a region-selective microglia activation, impaired hippocampal-depen-dent memory and altered behaviorally-induced expression of the immediate early gene Arc [234]. A therapeutically relevant dose of memantine (10 mg/kg/day s.c. for 28 days) was also able to restore *Arc* gene expression to normal levels, reduce the number of activated microglia an expression of brain inflammation - and ameliorate spatial memory impairments [234]. These results confirm the role of dynamic changes in Arc expression in neuronal plasticity and demonstrate the ability of memantine to reinstate the dynamic balance of cellular processes that were disturbed by chronic brain inflammation.

##### β-Amyloid

Chronic s.c. osmotic pump infusion of memantine (steady-state plasma concentrations of 2.34 µM, for 9 days) reduced local neuronal cell loss produced by intra-hippo-campal injection of Aβ_1-40 _[172]. Recently, the same group reported inhibition of apoptosis induced by Aβ_1-40_ infused in the hippocampus by memantine [15, 173].

Neuroprotective effects of memantine against Aß toxicity have also been shown in functional terms. Indeed, it was recently shown that infusion of memantine prevented the development of delayed non-matching to sample lever pressing task impairments produced by infusion of Aβ_1-40_ in rats [301]. Rats injected in the hippocampus with aggregated Aß_1-40_ (4 µg/µl), and two days later, with the NMDA receptor agonist ibotenic acid (0.3 µg/0.5 µl) showed learning deficits in the Morris water maze task and neuronal damage in the hippocampus from 5 to 6 weeks after the injection [180]. Memantine (10, 20 mg/kg/day s.c. infusion for 6 weeks starting 24 h before the Aß_1-40_ injection) caused a significant inhibition of the learning deficits, but a lower dose of memantine (5 mg/kg/day) and both tested doses of MK-801 (0.312, 0.624 mg/kg/day) did not have significant effects. In naive rats, MK-801 produced a significant learning impairment in the water maze task at a dose of 0.624 mg/kg/day, whilst memantine (20 mg/kg/day s.c. infusion) did not [180].

In a murine transgenic model of AD (APP23), memantine treatment prevented the time-dependent decrease in performance seen in vehicle-treated animals. This “disease-modifying” effect was observed three weeks after treatment termination, an important control in such functional studies supporting neuroprotective effects in the absence of interference from potential symptomatic effects in the continuous presence of drug [278]. Also, transgenic mice (APPswe) treated chronically with memantine showed lower levels of membrane-bound APP [275].

In studies in cultured human neuroblastoma (SK-N-SH) cells, treatment with memantine for 24 to 48 hours provided evidence that the drug may decrease disturbed APP processing [142]. Memantine treatment of cultured human neuroblastoma cells and primary fetal rat cortical neurons, albeit for a longer duration of 12 days, also decreased levels of Aβ_1-40_ starting at a therapeutically relevant concentration of 1 µM [142].

Memantine (1µM), APV (10 µM) as well as soluble tumor necrosis factor alpha (TNFα) receptor protected neurons from Aß_1-42_ stimulation microglial-conditioned media-induced toxicity, implicating the excitatory neurotransmitter glutamate and the pro-inflammatory cytokine TNFα as effectors of microglial-stimulated death [73]. Interestingly, *in vitro*, brief exposure of cultured cortical neurons to memantine (1-20 µM), which would produce only a transient block of NMDA receptors, inhibited the toxicity of NMDA for up to 48 hours with an IC_50_ of around 5µM [272]. In the same study, a high concentration of MK-801 (1µM) provided long-term protection against toxicity due to NMDA, AMPA, staurospo-rine, etoposide, hypoxia and Aß_1-40_, applied 48 hours later, but the relevance of this for memantine with regard to chronic therapy in AD remains to be investigated [272].

Chronic exposure of hippocampal cultures to soluble Aß oligomers induced reactive oxygen species and produced abnormal spine morphology and a decrease in their density. Subsequent consequences of this, such as synaptic deterioration including loss of the synaptic spine cytoskeletal protein debrin, were completely prevented by memantine (5-10 µM) [123, 141].

Memantine (1-10 µM) did not protect against Aß_1-42_ toxicity in cultured rat septal cholinergic neurons, but this is not surprising, as these neurons themselves are relatively resistant to NMDA toxicity [122].

##### Tau

Overexpression of human tau and of some of its N-terminal fragments in primary neuronal cultures leads to an NMDA receptor-mediated, caspase-independent pathway that was completely prevented by treatment with memantine (10 µM) [7]. The authors proposed that “death” signalling likely originates from stimulation of extrasynaptic NR2B subunit-containing NMDA receptors and sustained phosphorylation of extracellular-regulated kinases 1 and 2. Moreover, NMDA receptor involvement causes the fatal activation of calpain, which, in turn, degrades tau protein into a 17-kDa peptide and possibly other highly toxic N-terminal peptides.

The activity of protein phosphatase (PP)-2A, which regulates tau phosphorylation, is compromised in the AD brain. Memantine (1µM) reduced phosphorylation of tau (ser262) and prevented neurotoxicity produced by okadaic acid in a rat hippocampal slice preparation [150]. Transient transfection of PC12 cells with inhibitor-2 of PP2A (I(2)(PP2A)) also causes abnormal hyperphosphorylation of tau at Ser396/ Ser404 and Ser262/Ser356. Memantine (10 µM) inhibited this abnormal phosphorylation of tau and cell death and prevented the I(2)(PP2A)-induced inhibition of PP2A activity with no effect on basal activity [47]. It is not clear whether this effect is solely due to NMDA antagonism, since a competitive antagonist and an antagonist acting at the glycine site of the NMDA receptor were ineffective, at least at the concentrations used (ibid.).

It has also recently been shown that after chronic treatment of AD patients with memantine for 12 months, the CSF levels of phosphorylated tau decrease while non-phosphory-lated tau and Aß remain unchanged [59b].

Recently, further insights into the possible MOA for this effect became available. It was reported that memantine caused an increase *in vivo* in phosphorylation of GSK-3, which inhibits its function and could thereby reduce phosphorylation of presenilin 1, 2 and tau [58], but it is very unclear whether this has anything to do with NMDA receptor blockade due to the very high dose used (50 mg/kg). 

Taken together, these findings suggest that memantine might also be useful for the treatment of related tauopathies.

##### Summary of Neuroprotection in AD

On the basis of the animal experiments detailed here and in many previous studies, memantine at therapeutic doses in AD patients [224, 271] could provide inhibition of disease progression due to its neuroprotective properties [196].

#### Neurorestorative Effect

Memantine enhanced neurogenesis at therapeutically relevant concentrations in cortical cultures *in vitro* (the best effects were seen at 1 µM, with 140% of control neurogenesis) and in the DG and forebrain sub-ventricular zone *in vivo* (7.5 mg/kg p.o. once daily for 14 days, around 126% of control neurogenesis) [112]. However, in another study, although acute treatment of rats with memantine (20 mg/kg) also led to an increase in neurogenesis in the hippocampus, this effect disappeared after 7 days. Moreover, in contrast to acute treatment, chronic infusion of memantine with mini-pumps (20 mg/kg/d) failed to increase neurogenesis [253].

## SECONDARY PHARMACODYNAMICS

As a reminder, this review is intended to provide an update on preclinical studies on the pharmacodynamics of memantine. For most studies prior to 1999, the reader is referred to a previous review [196]. Apart from studies which are supportive of the neuroprotective effects of memantine, new data on secondary pharmacodynamic effects are presented with no ordered preference for validity or relevance.

### Activity in Models of Neurodegenerative Conditions

#### Introduction

It is widely accepted that NMDA receptor antagonists have neuroprotective activity in a variety of animal models of acute excitotoxicity. In acute ischemia, they are generally more active in models of focal, rather than global ischemia, when confounding factors such as changes in body temperature are taken into account [35]. Concerning the acute neuroprotective effects of memantine, the literature before 1999 has been reviewed in detail by [196]. For the sake of completeness, we included this information in a somewhat abbreviated form to provide a preface for new studies.

#### General Neuroprotective Activity

##### In Vitro

Numerous studies indicate that memantine protects against the toxic effects of exogenous NMDA receptor agonist in cultured neurones with an IC_50_ close to 1µM [196]. More recently, memantine was shown to protect rat neurons in organotypic hippocampal slices from NMDA-induced excitotoxicity (IC_50_ around 1 µM) and was similarly protective in cerebellar granule cell cultures when assessed as cell death with H-33342, but was less potent on viability assessed with MTT (IC_50_ around 10 µM) [280]. In the same study, (+)MK-801 negatively interfered with neuronal migration after excitotoxic insult in cerebellar micro-explant cultures, since the directed movement of neurons is dependent on physiological NMDA receptor activity. In contrast, memantine caused significant recovery from these deficits providing further evidence for the ability of memantine to differentiate between physiological and pathological activation of NMDA receptors [280].

Memantine alone (0.05-2.0 µM) concentration-depen-dently attenuated staurosporine-induced caspase-3 activity and lactate dehydrogenase (LDH) release in hippocampal cultured neurons after 7 and 12 days *in vitro* but had no effect in control cultures. These effects of memantine were more pronounced in hippocampal cultures than in neocortical and striatal cultures, which can be explained by different expression patterns of NMDA receptor subtypes [111]. Another group claimed that a higher concentration of memantine (10µM) potentiated neuronal death caused by exposure to staurosporine (10-30 nM for 48 h) in cultured murine neocortical neurons, but in their hands, caffeine 1 mM also exacerbated this death, which somewhat questions the validity of the model used [257, 258].

Memantine and (+)MK-801 blocked strong hypoxia / hypoglycemia-induced suppression of fEPSPs in rat hippocampal slices with EC_50_s of 14.1 and 0.53 µM respectively [76]. Whereas memantine blocked this effect with a similar potency to at which it blocks the induction of LTP, (+)MK-801 was four times less potent in this model than against LTP [77]. The calculated relative therapeutic indices (IC_50_ LTP over EC_50_ hypoxia / hypoglycaemia) for memantine and (+)MK-801 were 0.82 and 0.28, respectively. These results show that, even in a severe model of hypoxia / hypoglycemia, moderate affinity channel blockers exhibit a better therapeutic index than high affinity channel blockers. The authors proposed that it is likely that in milder forms of pathology, the observed differences in the therapeutic indices remain the same but the absolute values can be expected to be higher. Similarly, memantine (10 µM) also reduced LDH release from hippocampal slices by 40% following 1 hour of oxygen glucose deprivation (OGD) plus reoxygenation for 3 hours when treatment was started 30´ prior to OGD and continued for the duration of the study [261].

Memantine also protected cultured cerebellar granule cells from apoptosis induced by OGD (IC_50_ around 10 µM) or treatment with the mitochondrial toxin 1-methyl-4-phenylpyridinium (MPP+) or nitric oxide (NO) donors (IC_50_ around 10 µM) [280]. A very high concentration of memantine (50µM) blocked NMDA receptor currents and completely protected against 3hrs of OGD in a human embryonic teratocarcinoma cell line (NT2) that expresses NMDA receptors (NR2B>BNR2C>NR1>NR2A) [83]. Unfortunately, lower concentrations were apparently not tested.

Memantine (i.p. 5-50 mg/kg; 4 h) markedly increased BDNF mRNA levels in the limbic cortex and this effect was more widespread and pronounced at higher doses [164]. The effects of memantine on BDNF mRNA were also reflected in changes in BDNF protein levels. Moreover, memantine induced isoforms of the BDNF receptor trkB. The authors suggested that the neuroprotective properties of memantine could be mediated by the increased endogenous production of BDNF in the brain.

Memantine was somewhat less effective in protecting against acute excitotoxic insult by 3-nitrophenol (3-NP) in cerebellar micro-explant cultures [280], but these experiments were after acute 3-NP (0.5 mM) treatment c.f. treatment with 3-NP (35 µM) for 7-12 days in other studies more relevant for chronic excitotoxicity [115] - see below. Memantine (10 µM) reduced depolarization-induced superoxide radical formation in hippocampal slices following acute exposure to high extracellular K^+^ (50 mM). The block was less pronounced than that seen with MK-801 (1µM), possibly due to the greater voltage-dependency of memantine and/or the very high concentration of MK-801 used [242]. Exposure of cultured cortical neurons to elevated extracellular K^+^ concentrations (25 mM) induced membrane depolarization and an increase in action-potential firing that was associated with increased neuronal cell death following long-term treatment (15 DIV) [110]. This excitotoxicity was prevented by the continuous presence of an extremely high concentration of memantine (50µM) but lower concentrations were not tested [110].

In an organotypic hippocampal slice model with more relevance for the chronic, low level excitotoxicity proposed to occur in e.g. AD, memantine was protective with IC_50_ of c.a. 1-2 µM against semi-chronic excitotoxicity (4-20 days) produced by low concentrations of 3-NP (35 µM) as measured as changes in LDH release [115]. In this study, memantine also reduced both the cytoskeletal damage and synaptic decline in a concentration-dependent manner. Phase contrast microscopy, propidium iodide fluorescence and cresyl violet staining confirmed memantine´s neuroprotective effects. Furthermore, the protected tissue exhibited normal neuronal density and morphology in major hippocampal subfields.

The toxic effects of the scrapie prion protein, PrP(Sc), and its peptide fragment, PrP106-126, can be blocked by memantine (10 µM), which was therefore proposed to be a promising approach to prevent neuronal death in Creutzfeldt-Jakob disease [179].

The combination of 10 nM memantine and clenbuterol was also reported to be very effective against staurosporine toxicity *in vitro*, but it is very difficult to reconcile this concentration with the MOA as an NMDA receptor antagonist [52]. It is conceivable that this was a typo, and in fact memantine 10 µM was tested – see [55, 203].

##### In Vivo

As noted above, acute ischemia models are generally of a severe nature and the doses of NMDA receptor antagonist required are therefore very high. It is likely that lower doses provide neuroprotection inhibiting the progression of chronic neurodegenerative disorders such as AD.

Chronic dietary intake of memantine (31 mg/kg/day) for 14 days prevented death, convulsions and hippocampal damage induced by i.c.v. quinolinic acid [120]. Memantine also significantly attenuated malonate-induced striatal lesions implicating utility in chronic neurodegenerative diseases associated with deficits in mitochondrial function [246].

Rats infused i.c.v. with quinolinic acid alone showed clear learning deficits in the T-maze whilst those infused in parallel with memantine (20 mg/kg/day s.c.) were able to acquire the task normally [176]. Similarly, a decrease in choline uptake sites (an indicator of the density of ACh terminals potentially relevant for AD) was seen in the cortex of animals treated with quinolinic acid but not in those receiving additional treatment with memantine. In this context, it is important that infusion of this same dose of memantine in normal rats had no effect on T-maze learning or LTP in hippocampal slices *ex vivo* [176].

##### Ischemic Tolerance

It has been known for several years that under certain conditions, a short ischemic/hypoxic episode or pharmacological blockade of mitochondrial oxidative processes [227a] can decrease the susceptibility of neuronal tissue to subsequent severe insult - a phenomenon called ischemic tolerance. This fact might be of particular clinical importance in situations where a danger of succeeding episodes after moderate ischemic insult can be anticipated, such as in vascular dementia (VD) or combined AD / VD.

Memantine (5 mg/kg) did not inhibit ischemic tolerance to global ischemia in gerbils* in vivo* [62]. In fact, subchronic 3 day infusion of memantine (30 mg/kg/day) significantly decreased neurodegeneration on its own and potentiated the neuroprotective effects of ischemic pre-conditioning [62]. In a similar manner, memantine (20 mg/kg/day for 3 days *via* Alzet minipumps) did not block 3-NP (20 mg/kg i.p.) - induced delayed ischemic tolerance in rat hippocampal slices* ex vivo* [75]. In contrast, 3 days pre-treatment with a high dose of (+)MK-801 (2 mg/kg/day) tended to reduce ischemic tolerance following 3-NP preconditioning.

As such, although NMDA receptors do seem to be involved in ischemic / chemical tolerance, semi-chronic pre-treatment with therapeutically-relevant doses of memantine does not block this phenomenon [75].

#### Models of Acute Excitotoxicity

##### Stroke

The “real” effective acute neuroprotective doses / concentrations in almost all reported preclinical acute, focal and global ischemia models were always higher than, or similar to, those producing other behavioral effects regarded either as positive (chronic neuroprotective, anticataleptic, or antinociceptive effects) or as side effects (amnesia, ataxia, stereotypy) [35, 40, 171, 196, 243]. This fits perfectly with the known voltage-dependency of memantine and assumed strong depolarization during such strong acute insults. These acute ischemia models have less direct quantitative relevance for judgement of potential neuroprotective activity that would be useful for the inhibition of progression of chronic neurodegenerative disorders such as AD but are still useful for qualitative comparison of neuroprotective efficacies / side effects of different classes of NMDA receptor antagonist [76]. 

Interestingly, there is one clinical study conducted on 140 stroke patients showing a beneficial effect of memantine given i.v. at 20 mg/kg for 5 days following stroke [66]. 

In more recent preclinical reports, memantine (10 mg/kg) reduced neurological deficits and infarct volume when applied 15 minutes after three hours of middle coronary artery (MCA) occlusion in a temporary focal cerebral ischemia model [89]. In addition, memantine attenuated brain edema formation and BBB permeability. Additionally, memantine (20 mg/kg once daily i.p. for 3 days, first treatment started 30 min after the induction of intracerebral hemorrhage - ICH) caused a 47% reduction of hemorrhage volume, coupled with an inhibitory effect on the tissue plasminogen activator/urokinase plasminogen activator and matrix metalloproteinase-9 level in a rodent model of ICH (stereotaxic intrastriatal administration of type IV bacterial collagenase). This memantine treatment was also found to reduce inflammatory infiltration and apoptosis and was also determined to induce functional recovery after ICH [145]. 

Memantine (20 mg/kg i.p. 30 mins post infarct followed by 1 mg/kg b.i.d. for 48 hours) reduced infarct volumes (37 mm^3^ c.f. 81mm^3^ for controls) in a photothrombotic model of ischemia / stroke [263]. In a similar treatment paradigm, memantine was also effective against 2 hours of MCA occlusion in spontaneously hypertensive rats [45, 60, 285]. Even this apparently relatively high dose of memantine was reported to have no negative effect on learning in the Morris maze whereas (+)MK-801 1 mg/kg blocked learning [262]. However, there were errors in the rationale for these experiments – the memantine group was only on twice daily maintenance doses of 1 mg/kg (the necessity of which for the neuroprotective activity was not demonstrated) at the time of memory testing 72 hours after the high dose 20 mg/kg bolus which clearly, and not surprisingly, provided protection against the acute insult.

In 7-day-old newborn rats, memantine (20 mg/kg) had long term functional neuroprotective effects against learning deficits in the Morris maze when administered immediately following global hypoxic-ischemic brain injury and assessed 3-4 weeks later [82].

In a rabbit acute embolic infarct model, bolus i.v. injections of memantine at 1 mg/kg and 10 mg/kg did not alter the P(50) value and memantine and a dose of 25 mg/kg was lethal (NB: P(50) value = the amount of microclots (in mg) that produce neurological dysfunction in 50% of a group of animals). However, slowly infused memantine (25 mg/kg over 60 mins) significantly and substantially increased the P(50) value even when started 60 min following embolization. Memantine administered 180 min following embolization also increased the P(50) value but the response was more variable [144].

Memantine (5 mg/kg) showed no neuroprotection to mitigate cerebral injury after hypothermic circulatory arrest in a porcine model [227b].

##### TBI

Memantine (10 and 20 mg/kg, i.p.) showed neuroprotection after traumatic brain injury (TBI) induced in adult rats with a controlled cortical impact device. TBI led to significant neuronal death in the hippocampal CA2 and CA3 regions (by 50 and 59%, respectively), by 7 days after the injury. Treatment of rats with memantine immediately after the injury significantly prevented the neuronal loss in both CA2 and CA3 regions [221]. Memantine (10 mg/kg, i.p. 15 min after trauma) also significantly reduced lipid peroxidation levels in a rat model of closed head trauma in rats [194].

#### Models of Chronic Excitotoxicity

In a murine model of amyotrophic lateral sclerosis (ALS, SOD1(G93A) mice), memantine treatment (10 mg/kg b.i.d. s.c. starting on postnatal day 70 until end point) caused a significant, but very moderate delay in disease progression and also moderately increased the life span [283].

#### Glaucoma

In macaque monkeys, systemic treatment with memantine 4 mg/kg/day p.o. for 16 months was both safe and effective for reduction of functional loss associated with experimental glaucoma - argon laser treatment of the anterior chamber angle, whilst having no effect on intraocular pressure (IOP). These functional effects were seen at 3 and 5 months but not 16 months after an elevation of the intraocular pressure [95-97]. Moreover, histological measurements showed that this memantine treatment still promoted retinal ganglion cell (RGC) survival even at the 16 month observation period [96]. Furthermore, memantine improved tomographic measurements of nerve head topography taken at 3, 5 and 10 months after an elevation of IOP. The same group and others [91, 97] could also show similar results in a rat model for experimental glaucoma, where systemic treatment with memantine (10 mg/kg/day) was associated with a significant reduction in glaucoma-induced loss of retinal ganglion cells.

Memantine 5 mg/kg i.p. following optic nerve crush in rats caused a two-fold increase in compound action potential amplitude and a 1.7-fold increase in survival of RGCs respectively, two weeks after injury [298]. Memantine (10 mg/ kg/day *via* osmotic minipumps for three weeks) reduced ganglion cell loss to 12% when applied immediately after insult in a chronic ocular hypertension model induced by laser photocoagulation of episcleral and limbal veins. The same treatment also prevented any further loss when started 10 days after the first laser photocoagulation [298]. Memantine (5 mg/kg) significantly reduced ganglion cell loss after optic nerve crush in rats and memantine (1 µM) blunted the rise in intracellular calcium seen after administration of glutamate (125 µM) to retinal ganglion cells *in vitro* [182]. Optic nerve crush in Long-Evans rats led to a rise in extracellular glutamate; this rise was blocked by treatment with memantine 2 mg/kg daily starting 7 days prior to injury and continuing for 7 days thereafter [281].

In a model of glaucomatous optic neuropathy in an experimental glaucoma model in the rat, systemically applied memantine was injected intraperitoneally just before trabecular photocoagulation and provided neuroprotection [91].

DBA/2J mice spontaneously developed essential iris atrophy, pigment dispersion and glaucomatous changes (including cupping) over time. These changes were prevented by twice daily treatment with memantine 5 mg/kg i.p. for 4, 6 or 9 months, with significant differences to control for the later two time points [245].

Memantine 1 mg/kg i.m. once daily for 8 weeks protected against experimental optic nerve ischemia induced by chronic endothelin-1 (0.1 µg/day for 8 weeks *via* minipumps to perineural region of the anterior optic nerve) in rabbits [121]. In another study, memantine (5 mg/kg, i.p.) given at the onset of ischemia protected the rabbit retina from changes in GABA and ChAT immunoreactivities and induced a recovery of the reduced β-wave of the ERG [192]. Oral memantine (20 mg/kg) was also neuroprotective in a model of acute retinal ischemia in rabbits. The effect was evident at later follow-up time points as assessed by functional measures, but not by retinal imaging evaluating anatomical hallmarks [38].

A very high concentration of memantine (20 µM but not 500 µM) also blocked the increased levels of CaMKII subunits induced by intravitreal injection of glutamate in the adult rat retina [140].

In summary, the neuroprotective effects of memantine seen in various animal models of glaucoma seem to be very reproducible and provide a sound basis for the belief that memantine could be clinically neuroprotective in various excitotoxic diseases. As such, the results from ongoing clinical trials in glaucoma are eagerly awaited as a kind of clinical proof of concept.

#### Huntington´s Disease

Following infusion of 3-NP (12 and 24 mg/kg per day over 28 days *via* osmotic minipumps) into the basal ganglia of 24- to 28-month-old female rats, NMDA receptor antagonists, including memantine (24 mg/kg/day also *via* osmotic minipumps), were ineffective [108], or even increased neurodegeneration. An explanation for this contra-intuitive observation offered by the authors was the hypothesis that progressive neurodegeneration induced by low levels of energy may involve NMDA receptor-dependent protection of cells from caspase-mediated apoptosis and enhancement of mRNA levels of neurotrophins in the injured brain. They proposed that prevention of neurotrophin synthesis and the induction of caspase activity by NMDA antagonists may be deleterious for neuronal survival following injuries. However, in internal studies comparing the same age, strain and gender of rats used in this study with young male rats usually used for pharmacodynamic studies, plasma levels of memantine were 4.4 ± 1.6 µM versus 1.3 ± 0.24 µM with the same dosing regime (see below). As such, interpretation of the potential therapeutic consequences implied by the conclusions of this study is problematic. However, in a recent study, it has been shown that memantine (0.05–2.0 μM) did not induce any cytotoxic effect but attenuated the stauro-sporine-induced caspase-3 activity and LDH release in hippocampal cultured neurons *in vitro* [111]. The mematine-induced neuroprotection was more efficient in the hippocampal neurons than in the neocortical and striatal ones, which points to tissue specificity of effects of this neuroprotectant.

A small clinical study in Huntington’s patients already gave some indication for the neuroprotective activity of memantine in the clinical setting [16] giving a very good reason to follow this indication from a preclinical perspective.

#### Multiple Sclerosis

Semi-prophylactic administration of a very high dose of memantine (60 mg/kg p.o. from day 7 post-inoculation (PI)) significantly restored BBB integrity, reduced symptoms, and limited inflammatory lesions in a rodent of multiple sclerosis - experimental allergic encephalomyelitis (EAE) in Lewis rats - when assessed 12 days PI [204]. In this study, therapeutic application of memantine was found to be as effective as semi-prophylactic dosing [204]. It should be stressed that the doses used in this study were very high.

#### HIV Dementia

In an animal model of human immunodeficiency virus type 1-associated (HIV-1-associated)dementia - severe combined immuno-deficient (SCID)mouse model of HIV-1 encephalitis (HIVE) – memantine (5 mg/kg, i.p. daily for 7 days) reversed the deficits observed in synaptic transmission and long-term potentiation (LTP)in the CA1 region of hippocampal brain slices *ex vivo* [8]. *In vitro* neurotoxicity of the HIV-1 proteins Tat and gp120 was completely blocked by memantine (2 µM) [183], presumably by lowering gp120-induced increase Ca^2+^ concentrations in neurons [103].

#### Lupus

The autoimmune antibody R4A present in Lupus patients binds to the channel mouth domain of NR2A and NR2B and increases NMDA receptor EPSCs in a voltage-independent manner [106]. In this study, the antibody occluded LTP without affecting PPF or AMPA receptors and was toxic and caused memory deficits. These effects provide strong support for the signal-to-noise hypothesis for NMDA receptor dysfunction in synaptic plasticity. Indeed, memantine 5 mg/kg i.p. 30 minutes prior to LPS prevented hippocampal damage in a murine model of lupus (MAP-peptide immunization followed by LPS administration) [132].

#### Chemotherapy

New data indicate the potential of memantine for the prevention of cognitive impairment produced by cancer therapy treatment. The effect of vincristine - (known to damage neurons), cytarabine –(known to produce damage in the cerebellum) and L-asparaginase– (known to be toxic through production of ammonia) were studied in rats in tests for exploration/cognition such as the hole board and water radial maze. In all models, clear impairment was noted which was prevented by chronic (14 days) treatment with memantine (10 mg/kg/day) when the test was done 24h after the last memantine dose [276].

### Symptomatological Activity

#### Pain

##### Inflammatory Pain

Memantine (1 mg/kg i.p.) was very effective (75% reduction) in blocking the second phase of pain related behaviour following i.pl. injection of formalin in mice whilst having no effect on motor coordination in the rotarod test [20]. In contrast, although memantine suppressed biting / licking behaviours in the formalin test in rats at 10 and 30 mg/kg there was no effect at 3 mg/kg or on flinching behaviours [241]. These same doses also reduced the degree of paw swelling in response to i.pl. formalin [241]. In relation to this finding, although only tested at therapeutically irrelevant doses, memantine inhibited the proliferation of synoviocytes *in vitro* with an IC_50_ of 65µM [195]. In studies from a different group using the same model in mice [279], memantine only preferentially inhibited the late phase at a very high dose of 20 mg/kg i.p. – this clearly contrasts with a relatively large literature and the additional studies cited above. However, in [279] memantine produced a partial reversal of both thermal and mechanical hyperalgesia in rats (minimum effective dose (MED) of 10 and 15 mg/kg i.p., respectively) following intraplantar injection of carrageenan [279].

In rats, very high therapeutic doses of memantine (21.5 to 46.4 mg/kg i.p.; ED_50 _35.2 ± 4.7 mg/kg at the peak effect) dose-dependently increased the mechanical nociceptive threshold in a model of inflammatory pain (i.pl. carrageenan) and inhibited single motor unit wind-up evoked by noxious electrical stimulation [46]. Injection of memantine (0.1 mg, 0.2 mg, and 1 mg) into the knee joint immediately before carrageenan injection (2%, 40 µL), but not when administered i.p., significantly prevented pain-related behaviours in rats [308]. The intra-articular injection of memantine (0.2 mg) also suppressed c-Fos expression in the laminae I-II and laminae V-VI at the L3-4 spinal level and the degree of the spinal c-Fos expression was correlated with the extent of the pain-related behaviour [308].

##### Neuropathic Pain

In a model of diabetic neuropathy, memantine (15 mg/kg i.p.) produced a symptomatic antinociceptive effect, whereas the “gold standard” gabapentin (100 mg/kg p.o.) had no significant effect [279].

Memantine showed dose-dependent (1.8-17.8 mg/kg) symptomatic, anti-allodynic activity in the Bennett model of chronic constriction injury (CCI) [161, 170] but there was little separation between anti-allodynic and rotarod effects. Co-administration with morphine or clonidine, each at 6 fixed equi-effective dose ratios, was not able to reveal any supra-additive i.e. synergistic effects (isobolographic analysis) [161]. In contrast, in this same sciatic nerve injury model, memantine had no symptomatic effects against either cold and mechanical allodynia – it should be noted that gabapentin was also inactive against mechanical allodynia in this study, but was active against cold allodynia [279].

Spontaneous ongoing single unit activity of spinal cord neurones in anesthetized rats two weeks following L5/6 spinal nerve ligation showed high sensitivity to gabapentin (s.c.) and morphine (i.t.) administration, being significantly reduced in a dose-depen-dent manner [266]. In contrast, morphine administered *via* the systemic route (1, 3 and 6 mg/kg i.v.) produced only modest and non-significant reductions of spontaneous activity. Similarly, memantine (1, 5 and 20 mg/kg i.v.) produced only minor effects at doses known to be effective on wind-up in a very similar model from the same group [267].

Intrathecal injection of memantine (300 nmol) significantly inhibited CCI-induced mechanical allodynia [181] although this route of administration is very unlikely to have any therapeutic relevance for memantine.

##### Visceral Pain

[190] explored the role of NMDA receptors in processing acute visceral noxious input, compared with somatic noxious input. Cardiovascular responses to graded uretal distensions or graded pinch stimuli to one hind-paw were inhibited by memantine (4-32 mg/kg, i.v.) with an ID_50_ of 14.5 ± 1.3 mg/kg, but these results were concluded to be due to a non-specific action. Similarly, a high intravenous dose of memantine (16 mg/kg) inhibited nociceptive responses of neurones in the L6-S2 spinal dorsal horn evoked by urinary bladder distension to 27% of control [41]. This effect was selective for neurones subject to inhibition from non-segmental cutaneous inputs (type I neurons).

Behavioural pain responses to visceral noxious mechanical stimulation were inhibited in a reversible, dose-dependent manner by intravenous administration of memantine (1-10 mg/kg, i.v.) [168]. Single fibre recordings of decentralized pelvic nerves showed that colorectal distension responsive afferent nerve activity was also inhibited by memantine [168]. Memantine i.v. dose-dependently attenuated the action potential firing of vagal afferent fibres innervating the rat stomach response to antral distension in anesthetized rats with an ID_50_ of around 2.5 mg/kg indicating possible peripheral antinociceptive therapeutic utility in visceral pain [248]. In contrast, memantine (10–30 µM) did not affect spontaneous or stretch-evoked firing rate of guinea pig oesophageal or rectal mechanoreceptors recorded extracellularly *in vitro*, whereas some inhibitory effect was seen with very high concentrations (100-300 µM) [302, 303].

##### Opioid Tolerance

Morphine analgesia in the formalin test was increased by memantine (5mg/kg i.p.) [249]. Pretreatment with memantine (2.5-15 mg/kg) also significantly and dose-dependently potentiated morphine-induced, but not fentanyl-induced [213], acute antinociception in the tail flick test [134, 212, 213]. Following an acute morphine challenge, memantine also enhanced morphine antinociception when tests were conducted 120 but not 30 minutes post-mor-phine [18]. In contrast, others reported that memantine (3 and 10 mg/kg i.p.) had no effect on acute morphine or fentanyl analgesia in the same model [223] – drugs effects averaged over 0.5 to 4.5 hrs.

All of these findings might indicate that the apparent synergistic antinociceptive effects could depend on the duration of the pain and the pharmacokinetics of the opioid – morphine has a much longer *in vivo* half life than fentanyl – and the associated development of rapid tolerance [136]. Indeed, confirmation for the prevention of morphine tolerance was seen following repeated administration of low-dose morphine (5 mg/kg, 8 days, once a day) - tolerance to morphine analgesia was prevented by memantine (10 and 30 mg/kg) but not dizocilpine (1 mg/kg) [61]. Memantine (3 or 10 mg/kg) treatment also prevented morphine tolerance in the tail-flick test in pro-oestrous female rats and in ovariectomized rats receiving 17-beta-estradiol [252]. Similarly, memantine treatment inhibited both the acute antinociceptive effect of morphine and reversed morphine tolerance in morphine-tolerant mice [214]. 

Memantine (7.5 mg/kg but not 2.5 mg/kg) as well as the metabotropic glutamate receptor type 5 antagonist MPEP (30 mg/kg but not 10 mg/kg) attenuated the development of tolerance to morphine-induced acute thermal antinociception in mice. When given together, the low doses of MPEP (10 mg/kg) and memantine (2.5 mg/kg) also significantly attenuated opiate tolerance. None of the treatments with glutamate antagonists produced antinociceptive effects or significantly affected acute morphine-induced antinociception in the tail flick test [135].

The antinociceptive effects of the peripherally restricted opioid agonist loperamide (3-10 mg/kg) in the formalin test were enhanced by memantine (3 mg/kg) but not by NMDA receptor antagonists that don´t penetrate the blood brain barrier suggesting that central NMDA receptor blockade may be necessary to enhance analgesia induced through peripheral opioid mechanisms [250].

#### Drug Dependence

##### Opioids

In accordance with previous literature, memantine (10 mg/kg or 46.3 µmol/kg, i.c.v.) inhibited naloxone-induced jumping in morphine-dependent mice [286]. Memantine attenuated (5 mg/kg) and even completely blocked (10 mg/kg) the expression of withdrawal-potentiated startle during naloxone (2.5 mg/kg)-precipitated withdrawal from a single dose of morphine sulphate (10 mg/kg). Memantine (2.5 or 5 mg/kg) also blocked expression of withdrawal-induced hyperalgesia [98] and the occurrence of withdrawal-induced aggressive behaviour at doses of 1-30 mg/kg [264].

Memantine (5 and 10 mg/kg) inhibited both the acquisition and expression of conditioned place aversion (CPA) during withdrawal in morphine-dependent rats and also reduced the intensity of the physical signs of withdrawal. When morphine was co-administered with memantine, the animals did not develop CPA and presented less intensity in the physical signs of morphine withdrawal [159]. However, in another study where morphine (20 mg/kg) pre-treatment significantly potentiated the ability of naloxone (0.01-0.3 mg/kg) to produce place aversion in mice [31], this effect was attenuated by D-CPPene (1 and 3 mg/kg) but not by memantine (1-10 mg/kg).

Memantine (2.5-40 mg/kg) produced neither place preference nor place aversion, but the higher doses of memantine tested (20 and 40 mg/kg) were able to completely block morphine-induced conditioned place preference [225]. Memantine (7.5 mg/kg i.p.) treatment during extinction conditioning abolished the ability of drug-related cues (priming dose of morphine) to evoke reinstatement, suggesting that this NMDA receptor antagonist can be useful in preventing relapse in opioid dependent individuals [216]. Similar effects were seen with higher doses (20 and 40 mg/kg) in a murine model of reinstatement behaviour [226].

In addition, memantine (0.3-10 mg/kg) suppressed the self-administration of morphine (0.125-4.0 mg/kg) in rats, whereas MK-801 (0.1 mg/kg) was ineffective [247].

However, memantine (7.5 mg/kg) inhibited the expression of place preferences conditioned with both morphine and sexual encounter, but had no effects in food-conditioned mice [215, 217]. Additionally, memantine (1, 3 or 10 mg/kg) dose-dependently reduced the expression of acute defensive behaviours (assessed using the social interaction paradigm) while increasing motor activity during caffeine withdrawal in mice [265]. These findings suggest that the effects of NMDA receptor blockade may not be limited to abused drug-reinforced behaviours [217]. Since memantine administration produced an antiaggressive effect only at doses that affected locomotion (20 and 40mg/kg), it is unlikely that the glutamatergic system mediates the antiaggressive actions of morphine [228].

There are also positive clinical data which are beyond the scope of this review to discuss in detail [25, 137].

##### Cocaine

Memantine (10 and 40 mg/kg) reverted cocaine-induced social withdrawal and the increase in avoidance and flight / escape in paired male mice [153]. Acquisition and expression of cocaine-induced place preference was prevented by memantine (7.5 mg/kg, i.p.) but not by the partial glycine_B_ site agonist ACPC (50 mg/kg, i.p.) [131].

Memantine (1-10 mg/kg) attenuated expression of cocaine-conditioned motor activity at doses that did not significantly affect spontaneous motor activity [23] and reversed behavioural sensitization, an animal model for the intensification of drug craving in cocaine addiction [151]. An effective combination was the administration of memantine with the D_2_-preferring DA agonist pergolide (0.08 mg/kg). Reinstatement of cocaine-seeking behaviour may be disrupted by either D-CPPene (0.3-3 mg/kg) or memantine (1-10 mg/kg). However, their effects seem to depend on the reinstating procedure (cocaine injection versus presentation of cocaine-associated cues) and route of cocaine administration (systemic versus intracerebral), as well as upon their own motor stimulant effects [23].

Memantine (2.5-20 mg/kg i.p.) dose-dependently decreased cocaine self-administration in rats trained to self-administer cocaine (0.25 mg/infusion). Under a progressive ratio (PR) schedule, MK-801 (0.15 mg/kg i.p.) increased the number of cocaine infusions in a manner similar to increasing the unit dose of cocaine, suggestive of potentiation of cocaine reward. Conversely, memantine (10 mg/kg i.p.) produced rate-decreasing effects on the PR schedule [107]. Similarly, pre-treatment with memantine protected against cocaine-induced convulsions with an ID_50_ of 16mg/kg [33].

Memantine (0.3-3 mg/kg *via* i.v. infusion) was tested in Rhesus monkeys trained to press levers reinforced with either cocaine-associated stimuli or 30 µg/kg cocaine infusion. The results were taken to suggest that memantine may indeed attenuate the conditioned reinforcing effects of cocaine-associated stimuli, but may also increase levels of cocaine self-administration [185].

Amphetamine-induced increases in carrier-mediated dopamine release in slices of rat nucleus accumbens (NAcc) and this effect was reversed to control levels by memantine (1µM), but not by (+)MK-801 (0.1 and 1 µM). The authors ascribe this difference to a possible involvement of NR1a/ NR2D subunits in this tissue and suggest possible therapeutic relevance for treating drug dependence [56].

There are also clinical data indicating a lack of abuse liability in cocaine addicts which are beyond the scope of this review to discuss in detail [50, 282].

##### Ethanol

Acute administration of memantine (10 mg/kg i.p.) induced an upregulation of NR1-1/NR1-2 and NR2B protein levels in the hippocampus and cortex, respectively [219]. Interestingly, this modulation of subunit expression was similar to the anti-craving compound acamprosate. Additionally, ethanol (50 mM) - induced upregulation of NMDA receptor protein (NR1, NR2A and NR2B) in cultured rat hippocampal neurons, was completely prevented by memantine (10 µM for 5 days; [160]).

Withdrawal from chronic (6 month) alcohol ingestion produced robust learning deficits in rats in the Morris water maze. Treatment with memantine starting at the beginning of the withdrawal phase (20 mg/kg bolus followed by 1 mg/kg every 12 h for 4 weeks) resulted in a complete reversal of these behavioural impairments [158]. In a similar model, memantine also dose-dependently (1-10 mg/kg, i.p.) suppressed ethanol withdrawal seizures [24, 130]. Repeated memantine administration decreased the acute motor impairing effects of ethanol [186].

Koros and collegues [125] assessed the effects of restraint stress and memantine (2.25 or 4.5 mg/kg) on the dose-response curve of ethanol discrimination. Stress did not alter the rate of responding. However, both doses of memantine tended to increase the rate of responding when given in combination with lower doses of ethanol (0.25-0.5 g/kg). In contrast, 4.5 mg/kg memantine decreased the response rate when combined with 1 g/kg ethanol. These results suggest that: (1) pre-exposure to acute restraint stress or memantine does not affect the dose-response curve of ethanol discrimination; (2) memantine given in combination with low doses of ethanol may enhance the discriminative stimulus of ethanol but inhibits at high ethanol doses. Accordingly, the memantine-induced decrease in experimental ethanol craving [104] may not be related to alterations in the cueing properties of ethanol. 

Memantine (13 mg/kg per day) also prevented the increase in the consumption of saccharin after a 1-week deprivation from free-choice, unlimited access to saccharin (0.1%, w/v) [306] indicating that the previously observed inhibitory action in a similar model following alcohol deprivation can be extended to other conditions of excessive drug/alcohol/ food intake. However, studies on the behavioural specificity of memantine on alcohol drinking in a schedule-induced polydipsia (SIP) task in C57BL/6J mice indicate that drug induced reduction in alcohol drinking was associated with other behavioural effects that included reduction in regulatory drinking, bringing into doubt the therapeutic possibility for ameliorating human alcohol addiction [68]. Finally, when administered concurrently with binge ethanol exposure, cannabidiol and antioxidants protected against hippocampal and entorhinal cortical neurodegeneration in a dose-dependent fashion whereas memantine (30 mg/kg/ day as 6*5 mg/kg) was without effect [94].

Nonetheless, there are some positive clinical data which are beyond the scope of this review to discuss in detail [26, 138].

##### Epilepsy

Memantine effectively prevented both clonic and tonic components of pentylenetetrazole-induced (35 mg/kg i.p.) kindling starting at a dose of 3 mg/kg i.p. [156]. Similar effects were seen against NMDA convulsions whereas ataxic side effects were first seen at doses some 6-fold higher [157, 235].

##### Anxiety

In our review from 1999 the available data did not give support for anxiolytic activity of memantine in animal models that could possibly be translated into efficacy in humans. Newer data have since been published that support this notion. Memantine (1.25 - 5 mg/kg i.p.) was also inactive in a different rat model of anxiety to those previously reported for memantine - the conditioned emotional response [175]. Moreover, in an illuminated platform avoidance task in rats, memantine (20 µg), injected into the ventromedial hypothalamus, actually increased anxiety [270]. Similarly, memantine 5 mg/kg/i.p. daily for 7 days had no effect on stress-induced increases in hippocampal nitrogen oxides in an animal model of post-traumatic stress disorder (sequential exposure of the animals to restraint stress, forced swim stress and finally halothane vapours, followed 7 days later with a brief re-stress forced swimming) [99].

However, memantine (4.0 and 8.0 mg/kg) did show anxiolytic effects in rats in the elevated plus-maze (EPM) in a study by [21] and these effects, as for other anxiolytic compounds tested, diminished after repeated testing exposure but not after repeated drug administration i.e. after adoption of an anxiolytic-insensitive behavioural strategy by the animals – the so called 'one-trial tolerance' phenomenon. Scopolamine (0.5-1.5 mg/kg) impaired the acquisition of this behavioural strategy to cope with the subsequent EPM exposure and thereby revealed the anxiolytic-like effects of memantine in a repeat trial [22]. 

#### Depression

Memantine produced a dose-dependent (2.5-15 mg/kg) antidepressant-like effect in the tail-suspension test; and the antidepressant-like effect of the highest memantine dose of 15 mg/kg appeared to persist with sub-chronic administration of memantine (3 days, twice daily) [126]. In the same study, when administered acutely 5 min before testing in the open field, memantine reduced rearing (1.875-30 mg/kg), ambulation (7.5 and 30 mg/kg) and grooming (30 mg/kg). As measured in three different tests, ataxia and stereotypy appeared only at the single highest dose of 30 mg/kg, 5 and 35 min after administration. In mice treated for 3 days, twice daily, the dose of 30 mg/kg continued to increase ambulation and decrease rearing and grooming, but no signs of ataxia and stereotypy were detected. These data indicate that different doses of memantine are required for therapeutic and side-effects, and that tolerance may develop to some of the side effects e.g. ataxia, but not to therapeutic effects e.g. antidepressant-like actions [126].

Memantine (1-3mg/kg) also produced antidepressant-like effects in the forced swimming test (FST) in mice that seemed to be mediated through an interaction with the l-arginine-NO-cGMP pathway [5] and dependent on the cellular signalling modulated by PKA, CaMKII and MAPK/ERK, but not by PKC [6].

Memantine (2.5 and 5 mg/kg) showed synergistic antidepressant activity when combined with imipramine, venlafaxine and fluoxetine in the FST in rats [232]. Moreover, memantine (10 mg/kg) prevented the development of hyperthermia and the increase in noradrenaline levels in the anterior hypothalamus following administration of serotonin (100 mg/kg) and clorgyline (2 mg/kg) – an animal model of serotonin syndrome, one of the most serious side effect of some classical antidepressants [188]. Combination of sigma ligands with memantine at low doses that were without effect on their own (SA4503 1-10 mg/kg; siramesine 1-3 mg/kg; 1,3 di-0-tolylguanidine (DTG) 2.5 and 5 mg/kg; memantine 2.5 mg/kg i.p.) also decreased immobility time in the FST in rats [255, 256]. This effect was antagonized by sigma receptor antagonists. 

However, memantine (10 mg/kg i.p.) had no effect on the CNS expression pattern of the vesicular glutamate transporter VGLUT1, upregulation of which has been proposed to be a useful marker for antidepressant activity [178]. Most importantly, in a small, double-blind, placebo-controlled clinical study, in 30 subjects with major depression, memantine (5-20 mg/day) (n = 14) for 8 weeks failed to show a significant antidepressant effect compared to placebo (n= 16) [307]. 

#### Parkinson´s Disease

As previously well documented, memantine was confirmed to block haloperidol-induced catalepsy starting at a dose of 3.5 mg/kg i.p. [236]. In control and MPTP treated mice, memantine (5 mg/kg i.p.) had no significant effect on dopamine or 3,4-dihydroxyphenyl-acetic acid (DOPAC) levels [84]. However, homovanillic acid in MPTP-treated mice was claimed to be increased by 300% and this effect was attributed to possible sparing of dopaminergic neurons – but there were some discrepancies in the tabular data (e.g. the methods state 5 mg/kg but the legends state 0.5 mg/kg) [84].

Intranigral administration of glutamate to rats with Parkinsonian syndrome induced by MPTP augmented the development of Parkinsonian symptoms (oligokinesia and muscular rigidity), but did not affect motor activity of intact animals [139]. Memantine administered i.p. in parallel with the induction of Parkinsonian syndrome weakened the development of oligokinesia and muscular rigidity in a dose-depen-dent manner starting from 5 mg/kg and abolished the toxic effect of glutamate [139]. 

In the substantia nigra and corpus striatum of reserpine-treated rats, acute injection of memantine (40 mg/kg) strongly increased L-DOPA decarboxylase (DDC), whilst not affecting or decreasing 5- HTPDC activity [72]. L-DOPA (25 mg/kg) on its own inhibited both enzymes in either brain region. The ability to increase the activity of DDC could be clinically important in the treatment of Parkinson’s disease, where the disappearance of DDC caused by the degeneration of nigrostriatal dopamine could contribute to the eventual loss of effectiveness of dopamine replacement therapy with L-DOPA.

One group suggested a putative benefit of memantine as an anti-Parkinsonian agent on the basis of its synergistic interactions with L-Dopa i.e. following co-administration of memantine (0.3 to 3 mg/kg) with sub threshold (5 mg/kg) or suprathreshold doses (20 mg/kg) of L-Dopa to hypokinesic drug-naïve or L-DOPA-tolerant MPTP-treated mice [12, 78, 79]; see also [13]). 

#### Tardive Dyskinesia

Memantine inhibited the development of haloperidol-induced persistent vacuous chewing movements (VCD) and attenuated the increase in preproenkephalin mRNA expression in a rat model of tardive dyskinesia, in which VCM were induced by 20 weeks of haloperidol administration [10].

#### Cancer

Even though the effective doses of memantine in this section are far beyond therapeutically relevant concentrations, we here summarize the potential effects of memantine against cancer for the sake of completeness.

When host rats were treated with very high doses of memantine (25 mg/kg twice daily, i.p.), an implanted glioma cell line secreting glutamate (RG2Glu+) responded with a small, but significant, decrease in tumour volume [269]. Memantine also restricted the growth of C6Glu+ tumours (subcloned C6 cells which also actively release glutamate).

On the other hand, *in vitro* memantine did not reduce proliferation of cultured C6Glu+ cells or RG2 glioma cells at therapeutically achievable concentrations although a significant reduction in proliferation of both cell types occurred at huge concentrations of 100-400 μM [269]. Memantine also inhibited *in vitro* growth of ten human cancer cell lines (four prostate, two breast and four colon) with half-maximal growth inhibition at very high concentrations of memantine (23 to 92 µM) [1]. Finally, a very high concentration of memantine (50µM) caused a rapid and reversible change in melanocyte morphology, which was associated with disorganisation of actin and tubulin microfilaments that could have implications in the treatment of melanoma [105]. In all cases, the concentrations of memantine required *in vitro* are probably far too high to be of any real therapeutic relevance.

The open question is therefore the disaccord between the *in vivo* and *in vitro* data. This could be explained by the theory that aggressive CNS tumours make “place” for their own expansion in the brain by releasing glutamate and destroying neurones *via* excitotoxic mechanisms.

#### Diabetes

Memantine (4 mg/kg/day for 8 weeks) prevented loss of gastric NO neurons and reduction of nNOS protein expression in an animal model of diabetes when administered 3 days after induction of diabetes with streptozotocin (STZ) [193]. Diabetes results in various biochemical abnormalities such as elevation of glutamate in the retina. Post mortem incubation of the retina of diabetic rats with memantine (30 µM) inhibited such elevations [133].

#### Other

The incidence of myocardial ischemia-induced ventricular tachycardia, ventricular fibrillation and mortality was not modified by treatment of rats with memantine (1.5 mg/kg), injected 5 minutes prior to occlusion. However, the incidence of ventricular tachy-cardia, ventricular fibrillation and mortality induced by myocardial ischemia-reperfusion was significantly reduced by memantine, and MK-801 and ketamine [53].

NMDA receptor channel blockade has also been tested on aggression in isolated male mice by the resident-intruder procedure [17]. Memantine (1-30 mg/kg) inhibited expression of aggressive behaviours only at doses that produced ataxia and thus, does not exert selective effects on aggression.

## SIDE EFFECTS

Memantine disrupted prepulse inhibition (PPI) in an acoustic startle gating test at high, supra-therapeutic doses (10 and 17 mg/kg i.p.) but not at lower doses of 1 and 3 mg/ kg. Furthermore, the magnitude of this disruption was reduced relative to high-affinity ligands like (+)MK-801 [295]. Another group showed that memantine (10 mg/kg) actually increased PPI at short prepulse-pulse intervals (10-20 ms), while inhibition was confirmed at longer intervals (60-120 ms). A higher dose (20 mg/kg) had only inhibitory effects. Ketamine produced similar effects but enhancement of PPI was less evident. The effect of memantine was attenuated by an atypical neuroleptic, quetiapine, but not by haloperidol, which seems to be characteristic for NMDA receptor antagonists. This group is planning a study of memantine in humans, where ketamine has an enhancing effect upon prepulse inhibition and a similar effect is expected from memantine [268].

Potential modifications of the behavioural profile of memantine following long-term administration was assessed by [101]. When memantine (20 mg/kg/day) was either infused or repeatedly injected for 14 days, tolerance was observed to the learning impairing and ataxic effects seen at acute high doses after both repeated administration and infusion. Sensitization to the locomotor stimulation was seen only following repetitive injections but not infusion of memantine, which is the therapeutically more relevant route of administration.

In organotypic hippocampal cultures chronic treatment with memantine at 1µM for 17-23 days had no effect on bicuculline seizures and actually increased cell death in the DG. Similar results were obtained with the competitive and glycine site antagonists APV (50µM) and DCKA (100µM). In contrast, the NR2B selective antagonist Ro-25 6981 (1µM) was protective following chronic, but not acute, administration. It should be noted that the chosen concentration for memantine was close to its therapeutic range whereas that of the NR2B antagonist was far above the IC_50_ for NR2B receptors [284].

In summary, the additional non-clinical studies conducted with memantine since our review in 1999 did not reveal any new signalss for potential side effects, drug-drug interactions or toxic changes attributable either to memantine or to it’s known degradation products for therapeutic doses relevant for the treatment of AD. 

### Abuse Potential

Memantine has been used in clinical practice for over 20 years, during which no cases of abuse have been reported. However, given the fact that memantine has partially overlapping pharmacology with some drugs that are indeed abused (mainly in the USA) such as ketamine or PCP, it is important to analyze data from animal models related to abuse potential of these agents. 

It should be stressed that, based on plasma levels, an acute dose of 5 mg/kg acute or s.c. infusion of 20 mg/kg/day is the maximum dose that can be considered therapeutically relevant in rats since such treatment leads to a ca. 1 µM plasma concentration which is the maximal concentration seen in humans treated therapeutically with memantine [55].

In a drug discrimination paradigm in rats based on lever pressing (indicative of subjective drug effects), memantine partially substituted for PCP first at a dose of 10 mg/kg but a 50 % decrease of the response rate was also seen at this dose which probably reflects myorelaxant activity [240]. In monkeys a similar picture was seen, i.e. memantine produced PCP-like responding only at doses of 5 mg/kg and above, i.e. at doses that produced a decrease in response rate and which are very high doses in this species [187]. In contrast, the high affinity antagonist (+)MK-801 caused substitution for PCP at doses which did not impair performance.

Also, in contrast to PCP, memantine did not substitute for cocaine [240]. However, when site-specific NMDA receptor antagonists were examined against intravenous cocaine self-administration (0.24 mg/infusion), memantine (2.5-20 mg/ kg, i.p.) and MK-801 (0.05-0.2 mg/kg, i.p.) dose-dependently decreased the number of cocaine infusions [107]. Antagonists at other NMDA receptor sites, L-701,324 (glycine_B_, 1.25-10 mg/kg p.o.) and CGP 39551 (competitive, 2.5-15 mg/kg, i.p.) were without effect. Memantine (0.3-3 mg/kg *via* i.v. infusion) was also tested in Rhesus monkeys trained to press levers reinforced with either cocaine-associated stimuli or 30 µg/kg cocaine infusion. The results were taken to suggest that memantine may indeed attenuate the conditioned reinforcing effects of cocaine-associated stimuli, but may also increase levels of cocaine self-administration [185].

Memantine produced only variable self administration in monkeys [187]. Memantine was 2-3 times less effectively self administered than PCP, and the effect was very variable depending on the monkey and the test day, and the maximum effect was very weak. According to the author's calculations, in order to obtain equivalent PCP-like intoxication in humans, a dose over 150 mg (p. o.) must be used, which is 7-15 times higher than the usual therapeutic dose. An important aspect is that all of the monkeys used in this study had an history of PCP or cocaine use which probably made them more prone for demonstration of self-administration of memantine. This claim is, in fact, supported by another unpublished study where pentobarbital was used to train the monkeys. All four rhesus moneys in the study did self administer pentobarbital at least 16 times a day for 3 consecutive days. When pentobarbital was replaced with memantine, the self-administration rate was similar to that of saline. Moreover, even forced administration of memantine (1mg/kg per infusion) did not initiate self administration.

Memantine did not produce place preference on its own, indicating that it has no reinforcing potential in this test [131, 211]. Consistently, dopamine levels were not affected in medial prefrontal cortex by an acute administration of memantine (20 mg/kg, i.p.) which also indicates that memantine is unlikely to possess psychotomimetic activity at therapeutically relevant doses [102].

In a self-stimulation test memantine, at doses up to 17 mg/kg, did not have a significant effect on the current frequency threshold for self-stimulation – only a slight increase was seen at 10 mg/kg. In contrast, (+)MK-801 decreased the threshold in a range of doses 0.1-0.4 mg/kg i.e. the current intensity that was previously not reinforcing, gained reinforcing value. This indicates substantial differences between different NMDA receptor channel blockers and demonstrates that memantine, in contrast to (+)MK-801, is most likely devoid of abuse potential [273, 274].

Chronic treatment with memantine did not result in sensitization of the behavioural response, in contrast to the other NMDA receptor antagonist (+)MK-801 [151].

In summary, most studies cited above indicate clearly that, at therapeutic doses, memantine has no abuse potential, but in contrast might actually inhibit abuse of addictive drugs such as morphine or ethanol (see above). The effects on cocaine abuse are more controversial as, in monkeys, memantine inhibited the reinforcing effects of conditioned stimuli but may have enhanced cocaine self-adminis-tration [185]. On the other hand, in rats, memantine produces rate “decreasing” effects under a progressive ratio of cocaine self-administration.

Therefore, although it cannot be completely excluded that memantine could be abused by a population with a history of abuse of drugs with overlapping pharmacology such as PCP or ketamine, this probably has little relevance for the treatment population i.e. AD patient.
